# No Race-Ethnicity Adjustment in CKD-EPI Equations Is Required for Estimating Glomerular Filtration Rate in the Brazilian Population

**DOI:** 10.1155/2020/2141038

**Published:** 2020-07-18

**Authors:** Amanda D. Rocha, Suzane Garcia, Andressa B. Santos, José C. C. Eduardo, Claudio T. Mesquita, Jocemir R. Lugon, Jorge P. Strogoff-de-Matos

**Affiliations:** ^1^Postgraduation Program in Medical Sciences, Fluminense Federal University (UFF), Niterói, Rio de Janeiro, Brazil; ^2^Postgraduation Program in Cardiovascular Sciences, Fluminense Federal University (UFF), Niterói, Rio de Janeiro, Brazil; ^3^Nephrology Division, Department of Medicine, Fluminense Federal University (UFF), Niterói, Rio de Janeiro, Brazil; ^4^Nuclear Medicine Division, EBESERH/Hospital Antonio Pedro, Fluminense Federal University (UFF), Niterói, Rio de Janeiro, Brazil

## Abstract

**Background:**

Glomerular filtration rate (GFR) is usually estimated from equations using serum creatinine (sCr), with adjustment for gender, age, and race (black or nonblack). The Chronic Kidney Disease Epidemiology Collaboration (CKD-EPI) is the preferred equation for adults, but it was validated for the United States population. We intended to evaluate if the race-ethnicity adjustment proposed for the sCr-based CKD-EPI equations is appropriate for the Brazilian population.

**Methods:**

CKD outpatients had blood samples collected for determination of sCr and serum cystatin C (sCys) levels. GFR was measured (mGFR) by plasma clearance of ^51^Cr-EDTA and used as the reference. We compared values of mGFR and estimated GFR (eGFR) by CKD-EPI equations based on sCr (eGFR_Cr_) and on the combination of sCr and sCys (eGFR_Cr-Cys_). For African Brazilian patients, eGFR was calculated either without or with race adjustment. Accuracy was considered acceptable if the difference between the values of eGFR and mGFR was ≤30% (P30).

**Results:**

100 patients were enrolled (58 ± 14 years, 46% male, 39% white and 61% African Brazilian). Mean mGFR was 46.7 ± 29.2 ml/min/1.73 m^2^. Mean eGFR_Cr_ and eGFR_Cr-Cys_ without race adjustment were 47.8 ± 30.1 ml/min/1.73 m^2^ and 46.4 ± 30.3 ml/min/1.73 m^2^, respectively. The corresponding P30 accuracy values were 79.0% and 83.0%. In the African Brazilian subgroup, values for mean mGFR and eGFR_Cr_ either without or with race adjustment were 49.8 ± 32.2 ml/min/1.73 m^2^, 50.4 ± 32.7 ml/min/1.73 m^2^, and 58.4 ± 37.9 ml/min/1.73 m^2^ (*P* < 0.001 vs. mGFR), respectively. P30 accuracy values for eGFR_Cr_ either without or with race adjustment were 75.4% and 67.2%, respectively.

**Conclusions:**

The use of CKD-EPI equations without race-ethnicity adjustment seems more appropriate for the Brazilian population.

## 1. Background

Glomerular filtration rate (GFR) is considered the best overall index of kidney function in health and disease [1]. GFR cannot be measured directly but can be assessed as urinary or plasma clearance of exogenous markers such as inulin, iothalamate, 51chromium ethylenediaminetetraacetic acid (^51^Cr-EDTA), technetium-labelled diethylene-triamine-pentacetate (^99m^Tc-DTPA), and iohexol [2]. However, these exogenous markers are impractical for routine clinical use because they are expensive and logistically demanding. In clinical practice, GFR is estimated by equations using serum levels of endogenous biomarkers such as serum creatinine (sCr) and cystatin C (sCys) [3]. Despite some limitations [1], sCr is the most commonly used biomarker for assessing GFR.

The sCr-based equations proposed by the Chronic Kidney Disease Epidemiology Collaboration (CKD-EPI) for adults are used worldwide and include a race-ethnicity adjustment for African Americans [4]. Such an adjustment factor was validated for the United States population [5]. However, recent studies suggest that the higher serum creatinine levels reported in blacks in the US and the consequent need for race-ethnicity adjustment in creatinine-based GFR equations may not be present in blacks from other countries [6, 7]. In Brazil, the use of race-ethnicity adjustment in the CKD-EPI equations is even more debatable due to the extensive miscegenation of the Brazilian population [8, 9].

Our study aimed to evaluate the appropriateness of the race-ethnicity adjustment of the sCr-based CKD-EPI equations for the Brazilian population.

## 2. Methods

### 2.1. Study Design

This is a cross-sectional study of the performance of CKD-EPI equations for estimation of GFR derived from serum creatinine (eGFR_Cr_) or combination of serum creatinine and serum cystatin C (eGFR_Cr-Cys_) in Brazilian patients with chronic kidney disease.

CKD-EPI equations for eGFR_Cr_ and eGFR_Cr-Cys_ calculation were employed with no race-ethnicity adjustment for white subjects, whereas African Brazilians had their eGFR calculated by the same equations, but with and without race-ethnicity adjustment (Table 1, Equations 1 and 2). As a reference, GFR was assessed by plasma clearance of ^51^Cr-EDTA and here referred to as measured GFR (mGFR).

A specific form with demographic, anthropometric, and clinical data was fulfilled in the day of GFR measurement. The local ethics committee approved the study. All participants signed the informed consent form.

### 2.2. Participants

All CKD patients older than 18 years registered at the Nephrology Division Clinics of the University Hospital were eligible to participate in this study. Exclusion criteria were pregnancy, liver cirrhosis, metastatic cancer, paraplegia, quadriplegia, or limb amputation.

### 2.3. Race-Ethnicity Classification

Participants were classified according to the three main race-ethnicity categories defined by the Brazilian Institute of Geography and Statistics: white, black, and mixed-race. The researcher in charge defined race-ethnicity according to the phenotypic appearance, on the day of GFR measurement. For this study, black and mixed-race subjects were classified into a single group and named as African Brazilians.

### 2.4. GFR Measurement

The procedures for the assessment of GFR started around 8:00 a.m. at the Nuclear Medicine Unit of the University Hospital. GFR was determined by ^51^Cr-EDTA plasma clearance using the “dose × decay/interception” method described by Brochner-Motersen [10] and modified by Brandström [11]. GFR was adjusted by body surface using Haycock's formula [12] and expressed in mL/min/1.73 m^2^.

First, a cannula was inserted into a forearm vein, and a 10 mL blood sample was obtained for sCr and sCys measurement. Next, ^51^Cr-EDTA (3.7 MBq, Amersham, United Kingdom) was injected in bolus (minute 0), and the cannula was flushed with saline and removed. Blood samples (10 mL) were collected in heparinized tubes from the contralateral upper limb peripheral vein at 150, 195, and 240 minutes for patients with estimated GFR ≥ 30 mL/min/1.73 m^2^ and 150, 195, 240, and 480 minutes for those with GFR estimated below such threshold. To define whether the duration of the evaluation would be 240 or 480 minutes, GFR was estimated by the CKD-EPI equation using the last sCr value available in the medical record.

Blood samples were centrifuged for 10 minutes at 3000 rpm and separated into two 2 mL plasma aliquots for ^51^Cr-EDTA counting. Radioisotopic counting lasted 30 minutes on the gamma counter (LB2111-SINASC Multi-crystal Gamma, Berthold Technologies, Bad Wildbad, Germany). The value for each point was defined by the average of the two aliquots counting.

### 2.5. Laboratory

sCr was measured in the Roche/Hitachi Cobas© system by the Jaffé method traceable to isotope dilution mass spectroscopy (CREJ2©, Roche Diagnostics GmbH, Mannheim, Germany). sCys was measured in the Siemens BN-II© analyser by nephelometry (Dade Behring©, Deerfield, IL, USA) and calibrated to the International Federation of Clinical Chemistry Working Group for Standardization of Serum Cystatin C (ERM-DA471/IFCC reference).

### 2.6. CKD Staging and Concordance between eGFR and mGFR

CKD staging based on GFR was defined according the Kidney Disease: Improving Global Outcomes (KDIGO) classification [4]: stage 1, ≥90 mL/min/1.73 m^2^; stage 2, 89 to 60 mL/min/1.73 m^2^; stage 3a, 59 to 45 mL/min/1.73 m^2^; stage 3b, 44 to 30 mL/min/1.73 m^2^; stage 4, 29 to 15 mL/min/1.73 m^2^; and stage 5, <15 mL/min/1.73 m^2^.

We also assessed the ability of each equation to correctly classify the patients in the same CKD stage defined by the measured GFR and expressed as the percentage of concordance.

### 2.7. Performance of the Equations

Equations for GFR estimation had their performance analysed according to bias, precision, and accuracy:

- Bias (absolute value) = estimated GFR − measured GFR
- Precision = median and interquartile interval of the difference between estimated GFR and measured GFR
- Accuracy (P30) = percentage of estimated GFR values with a difference equal to or lower than 30% of measured GFR

### 2.8. Statistical Analysis

Differences between measured and estimated GFR were analysed by the Bland–Altman method and expressed in graphs, with measured GFR on the abscissa and the difference (measured − estimated GFR) on the ordinate, in which negative values indicate GFR overestimation by the equation, whereas positive values mean underestimation.

Normality of data distribution was tested using the Kolmogorov–Smirnov test. Continuous variables are expressed as mean ± standard deviation in case of a normal distribution or as median (interquartile range), otherwise. Categorical variables are shown as frequencies.

Comparisons between the means were made by paired *t*-test. Frequencies were compared by Fisher's exact test. *P* values <0.05 were considered significant.

Analyses were performed using SPSS® software, version 18.0 for Windows (IBM©, Chicago, IL, USA).

## 3. Results

The general characteristics of the 100 participants are presented in Table 2. The mean age was 58 ± 14 years, and 46 were male. The most common causes of chronic kidney diseases were hypertension, diabetes, and chronic glomerulonephritis. Thirty-nine participants were classified as white and 61 as African Brazilians (27 black and 34 mixed-race).

In the whole group, mGFR was 46.7 ± 29.2 ml/min/1.73 m^2^. The eGFR_Cr_ with no race-ethnicity adjustment was 47.8 ± 30.1 ml/min/1.73 m^2^ (*P*=0.38 vs. mGFR), whereas eGFR_Cr_ including race-ethnicity adjustment was 52.7 ± 34.9 ml/min/1.73 m^2^ (*P* < 0.0001 vs. mGFR). The eGFR_Cr-Cys_ without race-ethnicity adjustment was 46.4 ± 30.3 ml/min/1.73 m^2^ (*P*=0.70 vs. mGFR), whereas eGFR_Cr-Cys_ including race-ethnicity adjustment was 48.8 ± 32.2 ml/min/1.73 m^2^ (*P*=0.54 vs. mGFR), Table 3.

In the subgroup of white participants, mGFR was 41.9 ± 23.4 ml/min/1.73 m^2^. The eGFR_Cr_ was 43.7 ± 27.7 ml/min/1.73 m^2^ (*P*=0.26 vs. mGFR), and eGFR_Cr-Cys_ was 41.6 ± 27.4 ml/min/1.73 m^2^ (*P*=0.85 vs. mGFR) (Table 3).

In the African Brazilians subgroup, mGFR was 49.8 ± 32.2 ml/min/1.73 m^2^, and it was not significantly different from the white participants. The eGFR_Cr_ without race-ethnicity adjustment was 50.4 ± 32.7 ml/min/1.73 m^2^ (*P*=0.74 vs. mGFR), whereas eGFR_Cr_ with race-ethnicity adjustment was 58.4 ± 37.9 ml/min/1.73 m^2^ (*P* < 0.001 vs. mGFR). The eGFR_Cr-Cys_ without race-ethnicity adjustment was 49.4 ± 31.8 ml/min/1.73 m^2^ (*P*=0.74 vs. mGFR), whereas eGFR_Cr-Cys_ adding race-ethnicity adjustment was 53.4 ± 34.4 ml/min/1.73 m^2^ (*P*=0.012 vs. mGFR) (Table 3).

CKD stages prevalence according to the mGFR were as follows: 9% in stage G1; 18% in stage G2; 19% in stage G3a; 17% in stage G3b; 24% in stage G4; and 13% in stage G5. There was no significant difference between white and African Brazilian participants, but the prevalence of patients with reduced GFR (<60 ml/min/1.73 m^2^) tended to be higher among whites (82.1% vs. 67.2%, *P*=0.11) (Table 4).

The rate of patients correctly classified into CKD stages, taking mGFR as reference, varied from 52.5% of concordance for African Brazilians using eGFR_Cr_ with race-ethnicity adjustment to 66.7% for whites using eGFR_Cr-Cys_ equation, without statistically significant difference (Table 4). For most of the patients who fell in a stage different from the one they were using the mGFR value, the shifts only occurred between neighbour stages. However, shifts skipping one stage did take place on four occasions when eGFR_Cr_ equation with race-ethnicity adjustment was used and one occasion each when every other formula was utilized.

**Figure 1:**
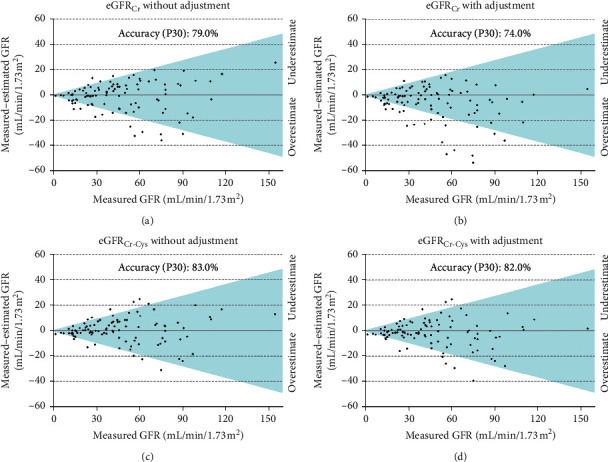
Accuracy of CKD-EPI equations for all participants (*n* = 100), in which dots inside the colour area mean a difference between measured and estimated GFR lower than 30% (P30).

The P30 accuracy of eGFR_Cr_ and eGFR_Cr-Cys_ equations for all patients with no race-ethnicity adjustment was 79.0% and 83.0%, respectively. When race-ethnicity adjustment was added in the equations, P30 accuracy of eGFR_Cr_ and eGFR_Cr-Cys_ was 74.0% and 82.0%, respectively (Table 5 and Figure 1).

**Figure 2:**
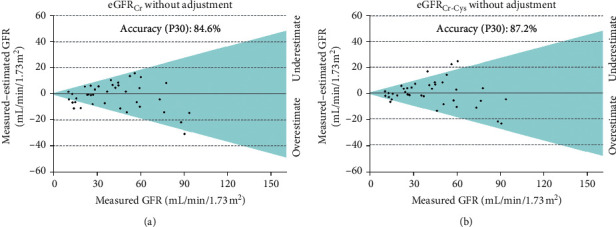
Accuracy of CKD-EPI equations for white participants (*n* = 39), where dots inside the colour area mean a difference between measured and estimated GFR lower than 30% (P30).

In the white participants' subgroup, P30 accuracy of eGFR_Cr_ and eGFR_Cr-Cys_ equations was 84.6% and 87.2%, respectively (Table 5 and Figure 2).

**Figure 3:**
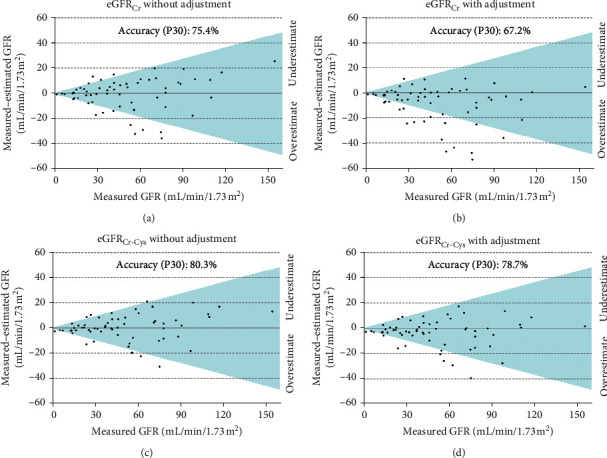
Accuracy of CKD-EPI equations for African Brazilians (*n* = 61), where dots inside the colour area mean a difference between measured and estimated GFR lower than 30% (P30).

In the African Brazilian subgroup, the P30 accuracy values for eGFR_Cr_ and eGFR_Cr-Cys_ equations with no race-ethnicity adjustment were 75.4% and 80.3%, respectively. When accounting for the race-ethnicity adjustment, P30 accuracy of eGFR_Cr_ and eGFR_Cr-Cys_ equations was 67.2% and 78.7%, respectively (Table 5 and Figure 3).

In the whole group, the precision of eGFR_Cr-Cys_ equations, without or with race-ethnicity adjustment, was higher than creatinine-only equations (Table 5). The absolute bias (mL/min/1.73 m^2^) of eGFR_Cr_ equation without race-ethnicity adjustment for all patients, whites, and African Brazilians were 0.1, 0.7 and −0.5, respectively. The correspondent bias (mL/min/1.73 m^2^) of the eGFR_Cr-Cys_ equation without race-ethnicity adjustment was −0.1, 0.4, and −0.2. After the inclusion of race-ethnicity adjustment, there was a significant increase in the bias of both eGFRCr and eGFRCr-Cys equations for all participants and the subgroup of African Brazilians (Table 5).

The performance of the equations was not significantly different when patients with mGFR <60 mL/min/1.73 m^2^ or ≥60 mL/min/1.73 m^2^ were studied separately. However, the P30 accuracy was numerically higher among participants with mGFR ≥60 mL/min/1.73 m^2^ for all equations (Table 6).

## 4. Discussion

Our study aimed to evaluate the performance of CKD-EPI equations for estimating GFR and the pertinence of race-ethnicity adjustment. To date, there have been no studies designed to answer such questions in the Brazilian population. Our findings suggest that the original CKD-EPI equations can be used in the Brazilian population, presenting an acceptable performance and a smaller bias if used with no race-ethnicity adjustment in African Brazilians.

Our findings are consistent with what we could expect if we critically review the history of the development of those equations in the United States and the description of their performance in other populations, such as sub-Saharan Africans.

Firstly, Levey et al. [13], in 1999, published the equations they developed from the Modification of Diet in Renal Disease (MDRD) study data. However, only patients previously diagnosed with CKD were enrolled in that clinical trial, and racial and ethnic minorities were underrepresented. In those equations, a race-ethnicity adjustment of 1.212 was added for blacks. Given the limitations of the MDRD equations, the Chronic Kidney Disease Epidemiology Collaboration consortium proposed new equations in 2009. Such new equations were developed and validated from data gathered from 10 different studies and were externally validated in participants from 16 other studies, including individuals with normal GFR and a greater racial and ethnic diversity [5]. Using the sCr-based CKD-EPI equations, the race-ethnicity adjustment for blacks was 1.159. Thus, for individuals of the same age, sex, and sCr, the estimated GFR will be 15.9% higher for blacks compared to individuals of other races and ethnicities. However, such race adjustment, in a binary fashion (black or nonblack), was validated only for the US population. Further studies suggest that the peculiarity of higher serum creatinine values found in African Americans and therefore the need to add a race-ethnicity adjustment may not be appropriate to blacks from other countries. Flamant et al. [14] studied 302 African Europeans who were pair-matched with white Europeans for mGFR (^51^Cr-EDTA renal clearance), age, gender, body mass index, and body surface area. They found that CKD-EPI equation with the African American race-ethnicity correction factor overestimated GFR in African Europeans. Nonetheless, African Europeans had serum creatinine 8% higher than their matched white Europeans, suggesting that a lower race-ethnicity correction is needed. In a recent study, Bukabau et al. [7] analysed the applicability of several estimating equations in sub-Saharan African populations. Using plasma clearance of iohexol as the reference, they found that both the Modification of Diet in Renal Disease and CKD-EPI equations performed better without the race coefficient. In fact, CKD-EPI equation without race adjustment and the Full Age Spectrum equation (FAS), a recently developed equation, which does not have a racial component, had the best performance in participants with normal GFR, whereas the FAS equation had a smaller bias and higher accuracy in those with GFR below <60 mL/min/1.73 m^2^.

In a previous study conducted in São Paulo, Brazil, the creatinine-based MDRD and CKD-EPI equations, with race-ethnicity and no race-ethnicity adjustment, were compared to the measured GFR by iohexol plasma clearance in 244 patients followed at a glomerulopathy outpatient clinic. The authors have found no increase in accuracy by adding race-ethnicity adjustment in the equations. However, only 8% of patients were African Brazilians [15].

Our finding of more unsatisfactory performance of equations by adding race-ethnicity adjustment indeed did not come as a surprise for us. In a previous study, our group analysed 1,303 individuals living in the city of Niterói, Rio de Janeiro, and 33% self-classified as white, 41% as brown, and 26% as black. In that analysis, we found no differences in serum creatinine values between the race groups, even after stratification by sex and age ranges, raising the question of whether adding race-ethnicity adjustment to eGFR equations would be appropriate for our population. However, the absence of measured GFR weakened our conclusions [9]. Those findings motivated the development of the present study so that we could confirm this hypothesis.

If the use of race-ethnicity adjustment had improved the performance of eGFR equations in our study, probably its routine adoption at the clinical practice in Brazil would not be an easy task. Brazilian population has an ethnic composition made up of an extensive mixture of three different ancestral roots: Europeans, sub-Saharan Africans and, to a lesser extent, Native Americans [8]. This mixture generated a considerable variability of skin pigmentation shade, with no discontinuity between dark and pale skin colour. Thus, the high degree of miscegenation in the population would make the adding of race-ethnicity adjustment to eGFR equations even more questionable.

The use of new equations that are not dependent on a race/ethnicity variable could solve this issue and be adopted worldwide and for different ethnical groups. The FAS equation seems a very promising alternative, since it is based on normalized SCr (sCr/*Q*), in which *Q* is the median sCr from healthy populations to account for gender and age. Thus, the FAS equation can be used indistinctly in children, adolescents, adults, and elderly, obviating the need of specific equations for each phase of life [16]. Later, new FAS equations were developed, incorporating cystatin C alone or the combination of sCr and cystatin C [17]. Even though FAS equations were validated for Caucasians only, the study of Bukabau et al. [7], with 494 participants from Democratic Republic of Congo and Ivory Coast, showed that both FAS and CKD-EPI equations without race-ethnicity adjustment had the best performance in those with normal GFR, whereas FAS equation had a smaller bias and higher accuracy in the individuals with GFR below <60 mL/min/1.73 m^2^.

Another critical issue that deserves discussion would be the necessity and convenience of developing specific eGFR equations for the Brazilian population. Our findings suggest that this would not be needed since the performance of the equations, with no race-ethnicity adjustment, was quite satisfactory, being just slightly more imperfect than that described in the original validation studies, with a little lower accuracy but no significant bias. The performance of creatinine-based CKD-EPI equations with no race-ethnicity adjustment was also similar to CKD-EPI equations using both sCr and sCys. Furthermore, the Brazilian population is substantially racially diverse, whereas countries in which specific equations were developed or adapted in order to improve the performance had a very homogeneous population, such as Japan [18]. Thus, we believe that the development of a bespoken equation for the Brazilian population only would be justified if there were a significant bias or the accuracy were unacceptably lower, which was not the case in our study.

Our study presents several limitations, including the small sample size and the geographical restriction of the studied population who probably is not representative of the whole Brazilian population.

In conclusion, the performance of CKD-EPI equations is acceptably good and can be used in the Brazilian population, and no race-ethnicity adjustment in creatinine-based CKD-EPI equations seems necessary and could indeed worsen the performance of the equations. Additional studies enrolling a broader number of patients are desirable to confirm our findings.

## Figures and Tables

**Table 1:** CKD-EPI equations for GFR estimating.

Equation 1: serum creatinine ([5])		
Gender	Creatinine (mg/dL)	
Male	≤0.9	GFR = 144 × (sCr/0.9)^−0.411^ × 0.993^age^ × (1.159 if black)
	>0.9	GFR = 144 × (sCr/0.9)^−1.209^ × 0.993^age^ × (1.159 if black)
Female	≤0.7	GFR = 141 × (sCr/0.7)^−0.329^ × 0.993^age^ × (1.159 if black)
	>0.7	GFR = 141 × (sCr/0.7)^−1.209^ × 0.993^age^ × (1.159 if black)

Equation 2: combination of serum creatinine and serum cystatin C ([3])		
Gender	Creatinine (mg/dL)	
Male	≤0.9	GFR = 135 × (sCr/0.9)^−0.207^ × (sCys/0.8)^*a*^ × 0.995^age^ × (1.08 if black)
	>0.9	GFR = 135 × (sCr/0.9)^−0.601^ × (sCys/0.8)^*a*^ × 0.995^age^ × (1.08 if black)
Female	≤0.7	GFR = 130 × (sCr/0.7)^−0.248^ × (sCys/0.8)^*a*^ × 0.995^age^ × (1.08 if black)
	>0.7	GFR = 130 × (sCr/0.7)^−0.601^ × (sCys/0.8)^*a*^ × 0.995^age^ × (1.08 if black)
		*a* = −0.375 if sCys ≤0.8 mg/L or −0.711 if sCys >0.8 mg/L

GFR, glomerular filtration rate; sCr, serum creatinine; sCys, serum cystatin C.

**Table 2:** General characteristics of the 100 participants.

Age (years)	58 ± 14

Male gender, *n* (%)	46 (46%)
Race-ethnicity, *n* (%)	
White	39 (39%)
African Brazilian	61 (61%)

Height (cm)	164 ± 9
Weight (kg)	74.9 ± 15.1
Body mass index (kg/m^2^)	27.3 ± 5.4
Serum creatinine (mg/dL)	1.61 (1.08–2.33)
Serum cystatin C (mg/L)	1.65 (1.11–2.45)

Primary renal disease, *n* (%)	
Hypertension	31 (31%)
Diabetes	19 (19%)
Chronic glomerulonephritis	13 (13%)
Lupus nephritis	9 (9%)
Chronic pyelonephritis	7 (7%)
Polycystic kidney	4 (4%)
Others	17 (17%)

Values are expressed as frequency, mean ± standard deviation or median (interquartile range).

**Table 3:** Measured and estimated GFR by CKD-EPI equations, with and without race adjustment.

	mGFR^51^Cr-EDTA	eGFR_Cr_ (no race adjustment)	eGFR_Cr_ (with race adjustment)	eGFR_Cr-Cys_ (no race adjustment)	eGFR_Cr-Cys_ (with race adjustment)
All participants (*n* = 100)	46.7 ± 29.2	47.8 ± 30.1	52.7 ± 34.9^*∗*^	46.4 ± 30.3	48.8 ± 32.2
White (*n* = 39)	41.9 ± 23.4	43.7 ± 27.7	—	41.6 ± 27.4	—
African Brazilian (*n* = 61)	49.8 ± 32.2	50.4 ± 32.7	58.4 ± 37.9^*∗*^	49.4 ± 31.8	53.4 ± 34.4^*∗∗*^

Values are expressed as mL/min/1.73 m^2^. mGFR: measured glomerular filtration rate; eGFR: estimated glomerular filtration rate; Cr: creatinine; Cys: cystatin C. ^*∗*^*P* < 0.001 vs mGFR; ^*∗∗*^*P* < 0.05 vs mGFR.

**Table 4:** Distribution of patients according to KDIGO classification of CKD staging, based on mGFR and eGFR, and the concordance rate of CKD staging between eGFR and mGFR.

Method of GFR analysis		All (*n* = 100)	White (*n* = 39)	African Brazilian (*n* = 61)
mGFR (^51^Cr-EDTA)				
CKD stage	G1	9 (9.0%)	2 (5.1%)	7 (11.5%)
	G2	18 (18.0%)	5 (12.8%)	13 (21.3%)
	G3a	19 (19.0%)	9 (23.1%)	10 (16.4%)
	G3b	17 (17.0%)	7 (17.9%)	10 (16.4%)
	G4	24 (24.0%)	11 (28.2%)	13 (21.3%)
	G5	13 (13.0%)	5 (12.8%)	8 (13.1%)

eGFR_Cr_ (no race adjustment)				
CKD stage	G1	12 (12.0%)	4 (10.3%)	8 (13.1%)
	G2	18 (18.0%)	5 (12.8%)	13 (21.3%)
	G3a	14 (14.0%)	4 (10.3%)	10 (16.4%)
	G3b	20 (20.0%)	10 (25.6%)	10 (16.4%)
	G4	25 (25.0%)	14 (35.9%)	11 (18.0%)
	G5	11 (11.0%)	2 (5.1%)	9 (14.8%)
Concordance with mGFR (%)		56.0 (46.2–65.3)	59.0 (43.4–72.9)	54.1 (41.7–66.0)

eGFR_Cr_ (with race adjustment)				
CKD stage	G1	17 (17.0%)	—	15 (24.6%)
	G2	16 (16.0%)	—	9 (14.8%)
	G3a	14 (14.0%)	—	10 (16.4%)
	G3b	20 (20.0%)	—	10 (16.4%)
	G4	26 (26.0%)	—	12 (19.7%)
	G5	7 (7.0%)	—	5 (8.2%)
Concordance with mGFR (%)		55.0 (45.2–64.4)		52.5 (40.2–64.5)

eGFR_Cr-Cys_ (no race adjustment)				
CKD stage	G1	10 (10.0%)	3 (7.7%)	7 (11.5%)
	G2	20 (20.0%)	6 (15.4%)	14 (23.0%)
	G3a	8 (8.0%)	2 (5.1%)	6 (9.8%)
	G3b	25 (25.0%)	10 (25.6%)	15 (24.6%)
	G4	29 (29.0%)	15 (38.5%)	14 (23.0%)
	G5	8 (8.0%)	3 (7.7%)	5 (8.2%)
Concordance with mGFR (%)		64.0 (54.2–72.7)	66.7 (50.9–79.4)	62.3 (49.7–73.4)

eGFR_Cr-Cys_ (with race adjustment)				
CKD stage	G1	14 (14.0%)	—	11 (18.0%)
	G2	16 (16.0%)	—	10 (16.4%)
	G3a	12 (12.0%)	—	10 (16.4%)
	G3b	22 (22.0%)	—	12 (19.7%)
	G4	29 (29.0%)	—	14 (23.0%)
	G5	7 (7.0%)	—	4 (6.6%)
Concordance with mGFR (%)		62.0 (52.2–70.9)		59.0 (46.5–70.5)

mGFR: measured glomerular filtration rate; eGFR: estimated glomerular filtration rate; Cr: creatinine; Cys: cystatin C. Frequencies are expressed by number (percentage), and concordance between CKD stages as percentage (95% confidence interval).

**Table 5:** Performance of the equations for estimation of GFR.

	eGFR_Cr_ (no race adjustment)	eGFR_Cr_ (with race adjustment)	eGFR_Cr-Cys_ (no race adjustment)	eGFR_Cr-Cys_ (with race adjustment)
Bias (mL/min/1.73 m^2^)				
All participants	0.1 (−6.5–7.1)	2.8 (−1.9–11.0)^a^	−0.1 (−6.2–4.0)	1.6 (−3.7–5.5)^b^
White	0.7 (−5.6–7.8)	—	0.4 (−5.6–4.8)	—
African Brazilian	−0.5 (−8.1–5.9)	3.2 (−0.5–14.3)^a^	−0.2 (−7.1–3.0)	2.4 (−2.2–5.8)^b^

Precision (mL/min/1.73 m^2^)				
All participants	6.8 (3.2–11.9)	6.3 (2.2–12.2)	5.1 (2.0–10.7)^c^	4.5 (2.3–10.3)^d^
White	6.3 (3.5–11.1)	—	4.8 (2.1–8.4)	—
African Brazilian	7.4 (3.0–13.3)	5.5 (2.0–14.3)	5.5 (2.0–11.4)	4.3 (2.4–11.8)

Accuracy (P30)				
All participants	79.0 (70.0–85.9)	74.0 (64.6–81.6)	83.0 (74.3–89.2)	82.0 (73.2–88.4)
White	84.6 (69.9–93.1)	—	87.2 (72.8–94.9)	—
African Brazilian	75.4 (63.2–84.6)	67.2 (54.7–77.7)	80.3 (68.5–88.5)	78.7 (66.7–87.2)

eGFR: estimated glomerular filtration rate; Cr: creatinine; Cys: cystatin C. Bias and precision are expressed as median (interquartile range), and accuracy as percentage (95% confidence interval). ^a^*P* < 0.0001 vs. eGFR_Cr_ without race adjustment and eGFR_Cr-Cys_ without race adjustment; ^b^*P* < 0.0001 vs. eGFR_Cr-Cys_ without race adjustment; ^c^*P*=0.013 vs. eGFR_Cr_ without race adjustment; ^d^*P*=0.005 vs. eGFR_Cr_ with race adjustment.

**Table 6:** Performance of the equations according to the range of measured GFR using the threshold of 60 mL/min/1.73 m^2^.

	eGFR_Cr_ (no race adjustment)	eGFR_Cr_ (with race adjustment)	eGFR_Cr-Cys_ (no race adjustment)	eGFR_Cr-Cys_ (with race adjustment)
GFR ≥ 60 mL/min/1.73 m^2^ (*n* = 27)				
Bias (mL/min/1.73 m^2^)	−4.0 (−11.3–14.6)	5.5 (−2.6–21.8)^a^	−3.6 (−11.8–10.2)	2.4 (−3.8–15.5)^b^
Precision (mL/min/1.73 m^2^)	12.1 (8.3–19.7)	11.6 (4.2–21.8)	11.0 (5.5–20.0)	8.7 (3.1–17.3)
Accuracy (P30)	85.2 (66.9–94.7)	77.8 (58.9–89.7)	85.2 (66.9–94.7)	88.9 (71.1–97.0)

GFR < 60 mL/min/1.73 m^2^ (*n* = 73)				
Bias (mL/min/1.73 m^2^)	0.1 (−4.9–6.4)	1.8 (−1.8–7.4)^a^	0.1 (−4.1–2.4)	1.5 (−3.6–3.7)^b^
Precision (mL/min/1.73 m^2^)	5.5 (1.6–9.2)	5.6 (1.8–10.5)	3.4 (1.7–8.2)	3.5 (2.2–7.8)
Accuracy (P30)	76.7 (65.7–85.0)	72.6 (61.4–81.6)	82.2 (71.7–89.4)	79.5 (68.7–87.2)

eGFR: estimated glomerular filtration rate; Cr: creatinine; Cys: cystatin C. Bias and precision are expressed as median (interquartile range), and accuracy as percentage (95% confidence interval). ^a^*P* < 0.0001 vs. eGFRCr without race adjustment and eGFR_Cr-Cys_ without race adjustment; ^b^*P* < 0.05 vs. eGFR_Cr-Cys_ without race adjustment.

## Data Availability

The datasets analysed during the current study are available from the corresponding author on reasonable request.
